# The low fresh gas flow anesthesia and hypothermia in neonates undergoing digestive surgeries: a retrospective before-after study

**DOI:** 10.1186/s12871-020-01140-5

**Published:** 2020-09-03

**Authors:** Yu Cui, Yu Wang, Rong Cao, Gen Li, Lingmei Deng, Jia Li

**Affiliations:** 1 Department of Anesthesiology, The Affiliated Hospital, School of Medicine, UESTC Chengdu Women’s & Children’s Central Hospital, No.1617, Riyue Avenue, Qingyang District, Chengdu, 610091 PR China; 2 Department of Anesthesilogy, No.363 hospital, Chengdu, 610041 China; 3 Department of Anesthesiology, Vanderbilt University Medical Center, Nashville, TN 37232 USA

**Keywords:** Low fresh gas flow anesthesia, Neonates, Hypothermia

## Abstract

**Background:**

Based on the previous investigation in our institution, the incidence of intraoperative hypothermia in neonates was high. Since September 1st, 2019, the recommendation had been launched to utilize ≤1 L/min fresh gas flow during the neonates’ surgical procedure. We therefore intended to evaluate the association between low fresh gas flow anesthesia and the occurrence of hypothermia in neonates undergoing digestive surgeries.

**Methods:**

A retrospective chart review, before-after study was conducted for neonates who underwent digestive surgeries. The primary outcomes were the incidence of hypothermia. The secondary outcomes included hospital mortality, the value of lowest temperature, blood loss, mean body temperature during the surgery, the length of hypothermia during the surgery and postoperative hospital length-of- stay (PLOS).

**Results:**

249 neonates fulfilled the eligibility criteria. The overall incidence of intraoperative hypothermia was 81.9%. The low fresh gas flow anesthesia significantly reduced the odds of hypothermia [routine group: 149 (87.6%) versus low flow group: 55 (69.6%); *p* < 0.01]. Moreover, the low fresh gas flow anesthesia could reduce the length of hypothermia [routine group: 104 mins (50, 156) versus low flow group: 30 mins (0,100); *p* < 0.01], as well as elevate the value of lowest temperature for neonates [routine group: 35.1 °C (34.5, 35.7) versus low flow group: 35.7 °C (35.3, 36); *p* < 0.01]. After adjustment for confounding, low fresh gas flow anesthesia and the length of surgical time were independently associated with intraoperative hypothermia.

**Conclusions:**

Low fresh gas flow anesthesia is an effective way to alleviate hypothermia in neonates undergoing open digestive surgery.

## Background

Intraoperative hypothermia that is defined as core temperature < 36.0 °C is one of complications faced by anesthesiologists during surgical period, which may exposure patients to acidosis, imbalanced oxygen consumption, delayed anesthesia recovery, coagulopathy, wound infections and bleeding [1, 2]. Available literature has proved that intraoperative hypothermia is an independent risk factor of early perioperative complications and mortality [3].

Among various surgeries, the occurrence of intraoperative hypothermia is high in digestive surgeries due to visceral and peritoneal surface exposure, anesthetic-induced impairment of thermoregulatory control and requirement of intestinal irrigation fluid. Lai et al. reported that patients’ age is one of main factors contributed to inadvertent intraoperative hypothermia, and the overall incidence is as high as 83.3% in neonates despite the passive and active temperature management have been conducted [4]. Some congenital intestinal disorders in neonates need urgent surgical intervention, i.e., necrotizing enterocolitis, meconium ileus, and congenital intestinal atresia. This is indeed a challenge for anesthesia providers and scrub nurses because normothermia is hard to be maintained on neonates undergoing digestive surgeries. Compared to adults, the neonates are more susceptible to develop hypothermia since the mechanism of thermoregulation has not yet been well established. This problem is amplified by inadequately warmed operating room and fluid infusion. With increasing awareness of intraoperative hypothermia, emerging evidence has proposed various methods to maintain normothermia [5], while the effectiveness strategies on neonates are limited.

Previous study had demonstrated that low-flow anesthesia technique was accepted by some anesthesiologists due to heat and humidity preservation, as well as decreased gas consumption [6]. Kleemann et al. found that the temperature of inspired gas during low-flow anesthesia was higher when compared to high-flow anesthesia, indicating heat could be well reserved by low-flow anesthesia [7]. However, that low fresh gas flow in sevoflurane anesthesia led to compound A generation was an issue worried by some anesthesia providers, since compound A had a dose-related nephrotoxicity which had been confirmed in laboratory test [8]. Even in the developed country, the target of fresh gas flow was 2 L/min for sevoflurane anesthesia [9]. Some anesthesia providers were reluctant to further reduce fresh gas flow during sevoflurane administration and utilized more than 1 L/min fresh gas flow to achieve desired anesthesia in clinical practice. In fact, as early as in 2000, Obata and colleagues had confirmed that prolonged low-flow sevoflurane anesthesia had the same effects on renal and hepatic functions as high-flow sevoflurane [10].

Based on the previous investigation in our institution, although the active temperature managements had been implemented, such as cotton blankets, mattress, and infusion fluid warming, the incidence of hypothermia in neonates reached up to 90%. To reduce the incidence of hypothermia in neonates, we hypothesized that low fresh gas flow anesthesia was a good way to keep normothermia in neonates. In our institution, till August 2019, the fresh gas flow to perform neonate’s anesthesia relied on the practitioner’s experience and opinion, and it might exceed 1 L/min. Since September 1st, 2019, the new recommendation had been launched to utilize ≤1 L/min fresh gas flow during the neonates’ surgical procedure. Therefore, we intended to evaluate the association of low fresh gas flow anesthesia with the occurrence of intraoperative hypothermia in neonates undergoing digestive surgeries.

## Methods

After obtaining Institutional Review Board (IRB) approval [No. 2020(3)], we retrieved patient data from the electrical record system at Chengdu Women’s and Children’s Central Hospital between June 31, 2018 (from the very beginning of electrical medical record utilization) and April 1, 2020. This study had been registered at http://www.chictr.org.cn/index.aspx with No. ChiCTR2000034242 on June 27, 2020. The necessity of informed consents was waived by the IRB, considering the nature of the retrospective study and the anonymized patient data.

### Study population

The inclusion criteria were neonates who were underwent digestive surgeries. The exclusion criteria include as follows: 1. Conversion from laparoscopic surgery to open surgeries; 2. Lacking documentation of temperature; 3. Congenital heart disease patients with clinical symptoms; 4. Digestive surgery were performed concurrently with other procedures.

### Data collection

Once the list of the patients was created, the following variables were collected from our electronic patient registration system, i.e., age at surgery, weight at surgery, sex, birth body weight, gestational days, ASA status, comorbidities, the length of surgical time, mean body temperature during the surgery, intraoperative flow of fresh gas, estimated blood loss, blood transfusion, plasma transfusion, fluid infusion during surgery, preoperative hemoglobin, postoperative hemoglobin, the duration of hypothermia, the lowest temperature, postoperative hospital length-of-stay (PLOS) and intraoperative urinary volume. Neonates were defined as patients under the age of 28 days. Mortality data was obtained from a combination of hospital discharge disposition, our death records and the legal guardian or power of attorney who withdrew treatment in the patients with endotracheal tube. Actual PLOS was from surgical date to discharge date. The duration of hypothermia was calculated from the beginning of hypothermia to the beginning of normothermia. As the rule in our institution, only anesthesia providers with 5 years’ experience were authorized to perform anesthesia in neonates. Since we had strict standard operative procedure guidelines, the strategies for avoiding hypothermia in our institution, included utilizing anesthesia station with heat and humidity exchanger, warm air circulation, warm infusion fluids, and warm mattress pads and blankets. Forced air warming was routinely used before and after the change in fresh gas flow practice. The temperature of the operating room was usually controlled between 22.0 °C and 24.0 °C. Dräger Primus anesthesia workstation with a heat and moisture exchanger was used for neonates. According to the description in background section, we therefore looked at the results from the patients that were underwent surgery before (Routine group) and after September 2019 (Low flow group).

### Intraoperative esophageal temperature

For neonates undergoing surgery under general anesthesia in our institution, an esophageal probe was inserted into esophagus after general anesthesia. Body temperature was automatically recorded at 10-min intervals until the patients left the operating room. Based on definition from several previous studies [1, 2], hypothermia was defined as body temperature < 36.0 °C and normothermia was considered as 36.0 °C ≤ body temperature ≤ 37.4 °C. When <36.0 °C was recorded on the electronic record, the patient was considered to have hypothermia. Intraoperative mean body temperature was defined as the sum of recorded body temperature divided by the total times. The esophageal probe was removed immediately before the patient left the operating room.

### Outcome measures

The primary outcome was the incidence of intraoperative hypothermia.

The secondary outcomes included.

1. hospital mortality,
2. the value of lowest temperature,
3. blood loss,
4. mean body temperature during the surgery,
5. the length of hypothermia during the surgery,
6. postoperative hospital length-of-stay (PLOS).

### Statistical analysis

The sample size was calculated based on the previous study done by Lo et al. [11] To detect a change in temperature of 0.5 °C with a study power of 90% and an alpha error of 5% the sample size required was 54. Considering 20% dropout because of lacking documentation of temperature, we recruited 65 patients in each group at least. The categorical variables were expressed as percentages. Continuous variables are presented as the mean ± standard deviation [SD] or median and interquartile range [IQR] (25–75%) if nonnormally distributed. The student t test was used to compare normally distribute data, otherwise the Mann Whitney U-test was used to compare two groups. The chi-squared test was used for categorical data. Multivariable logistic regressions were performed to determine the association between potential confounding variables and occurrence of intraoperative hypothermia. To adjust for confounding, analysis was performed using clinically relevant, the variables was selected in advance, including age, sex, weight at surgery, birth body weight, gestation days, the length of surgical time and fresh gas flow rate. These clinically relevant variables were selected based on biologic plausibility and were the most popular reported confounders in cohort on neonates [12]. Variance inflation factors for all predictors in multivariable regression were chosen to 1.0. Patients lacking documentation of temperature was excluded from the study. The analyses were carried out with R studio 3.5.2. The main R packages were listed in the additional file 1. *P* < 0.05 was statistically significant, and all tests were two-sided.

## Results

**Fig. 1:**
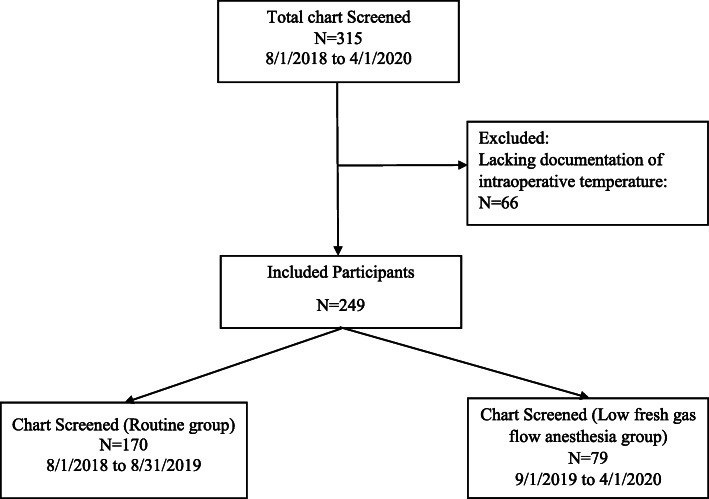
Study patient flow diagram

A total of 315 neonates were identified as having digestive surgeries during the study period. Of this group, 66 neonates were excluded from the study as lacking documentation of intraoperative temperature. After screening for inclusion and exclusion criteria, 249 patients enrolled in this retrospective study, including 170 who underwent routine fresh gas flow anesthesia group (routine group) and 79 in the low fresh gas flow anesthesia group (low flow group). All the patients underwent general anesthesia with tracheal intubation. (Fig. 1).

### Patient and clinical characteristics

Demographics data for both groups were presented in Table 1. The neonates in the two groups were demographically similar with the routine group composed of 98 boys (57.6%) and low flow group 50 boys (63.3%). At the time of surgery, body weight [2.7 kg (2.1, 3.2) in routine group and 2.9 kg (2.4, 3.2) in low flow group] and age [6 days (4, 13) in routine group and 6 days (3, 9) in low flow group] were comparable between two groups. There was no statistically significant difference detected between two groups with respect to gestation days, birth weight, ASA status, type of surgery, diagnosis, and surgical service.

**Table 1:** Baseline demographic characteristics of each group

	Overall patients (*n* = 249)	Routine (*n* = 170)	Low flow (*n* = 79)	*P value*
Age at surgery (days) (Median, IQR)	6.0 (4.0, 11.0)	6.0 (4.0, 13.0)	6.0 (3.0, 9.0)	*0.35*
Weight at surgery (kg) (Median, IQR)	2.8 (2.2, 3.2)	2.7 (2.1, 3.2)	2.9 (2.4, 3.2)	*0.27*
Sex (male, %)	148 (59.4)	98 (57.6)	50 (63.3)	*0.48*
Birth body weight (kg) (Median, IQR)	2.8 (2.1, 3.3)	2.8 (2.1, 3.3)	2.9 (2.2, 3.4)	*0.20*
Gestation days (Median, IQR)	264 (245, 274)	263 (242, 274)	265 (248, 274)	*0.83*
ASA, n (%)				*0.20*
I	1 (0.4)	0 (0)	1 (1.3)	
II	70 (28.1)	49 (28.8)	21 (26.6)	
III	159 (63.9)	111 (65.3)	48 (60.8)	
IV	19 (7.6)	10 (5.9)	9 (11.4)	
Type of surgery				*0.38*
Elective, n (%)	77 (30.9)	56 (32.9)	21 (26.6)	
Emergency, n (%)	172 (69.1)	114 (67.1)	58 (73.4)	
Diagnosis				*0.11*
Congenital anorectal malformation, n (%)	51 (20.5)	30 (17.6)	21 (26.6)	
Gastric perforation, n (%)	9 (3.6)	5 (2.9)	4 (5.1)	
Ileus, n (%)	10 (4.0)	7 (4.1)	3 (3.8)	
Intestinal atresia, n (%)	37 (14.9)	23 (13.5)	14 (17.7)	
Neonatal necrotizing enterocolitis (NEC), n (%)	117 (47.0)	90 (52.9)	27 (34.2)	
Pyloric hypertrophy, n (%)	5 (2.0)	2 (1.2)	3 (3.8)	
Umbilical bulge, n (%)	9 (3.6)	6 (3.5)	3 (3.8)	
Hernia, n (%)	1 (0.4)	1 (0.6)	0 (0)	
Diaphragmatic hernia, n (%)	3 (1.2)	2 (1.2)	1 (1.3)	
Appendicitis, n (%)	1 (0.4)	1 (0.6)	0 (0)	
Intestinal malrotation, n (%)	3 (1.2)	3 (1.8)	0 (0)	
Others, n (%)	3 (1.2)	0 (0)	3	
Surgical service				*0.08*
Anoplasty, n (%)	51 (20.5)	30 (17.6)	21 (26.6)	
Enterectomy, n (%)	164 (65.9)	120 (70.6)	44 (55.7)	
Gastric perforation repair, n (%)	9 (3.6)	5 (2.9)	4 (5.1)	
Pyloric myotomy, n (%)	5 (2.0)	2 (1.2)	3 (3.8)	
Umbilical bulge repair, n (%)	9 (3.6)	6 (3.5)	3 (3.8)	
Hernia repair, n (%)	1 (0.4)	1 (0.6)	0 (0)	
Others, n (%)	10 (4.0)	6 (3.5)	4 (5.1)	

### Primary and secondary outcomes

**Fig. 2:**
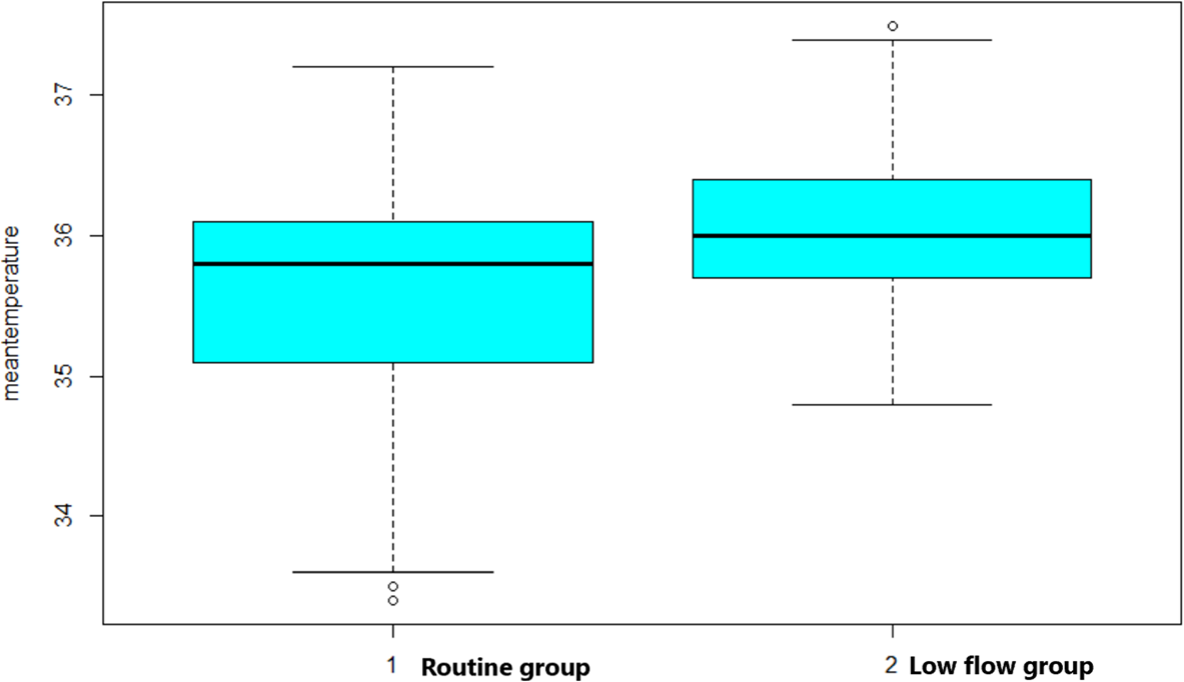
The meantemperature in Routine group and Low flow group. 1 = Routine group; 2 = Low flow group

Primary and secondary outcomes were reported in Table 2. In intraoperative period, the duration of surgery, blood loss, fluid infusion and pre−/post hemoglobin were comparable in both groups (Table 2). The overall incidence of hypothermia was 81.9%. Of the 249 patients in the cohort, the hypothermia occurred in 149 of 170 neonates (87.6%) allocated to routine group and in 55 of 79 neonates (69.6%) allocated to low flow group. The low fresh gas flow significantly reduced the odds of hypothermia [routine group: 149 (87.6%) versus low flow group: 55 (69.6%); *p*<0.01) and elevated the intraoperative mean temperature [routine group: 35.6 ± 0.8 °C versus low flow group: 36.0 ± 0.6 °C; *p*<0.01) (Table 2, Fig. 2). Moreover, low fresh gas flow could reduce the length of hypothermia [routine group: 104 mins (50, 156) versus low flow group: 30 mins (0,100); *p*<0.01], as well as elevate the value of lowest temperature for neonates [routine group: 35.1 °C (34.5, 35.7) versus low flow group: 35.7 °C (35.3, 36); *p*<0.01] (Table 2). No episodes of hypoxia or cardiac arrest were founded in our patients intraoperatively.

**Table 2:** Intraoperative and postoperative data

	Overall patients (*n* = 249)	Routine (*n* = 170)	Low flow (*n* = 79)	*P* value
Fresh gas flow rate (L/min) (Median, IQR)	2.0 (1.0, 2.0)	2.0 (2.0, 2.0)	1.0 (1.0, 1.0)	< 0.01*
Mean temperature during surgery (Mean ± SD)	35.7 ± 0.8	35.6 ± 0.8	36.0 ± 0.6	< 0.01*
Hospital mortality, n (%)	25 (10.0)	20 (11.8)	6 (7.6)	*0.43*
Hypothermia, n (%)	204 (81.9)	149 (87.6)	55 (69.6)	< 0.01*
Length of hypothermia (mins) (Median, IQR)	90 (20, 148)	104 (50, 156)	30 (0, 100)	< 0.01*
The lowest temperature (°C) (Median, IQR)	35.4 (34.7, 35.8)	35.1 (34.5, 35.7)	35.7 (35.3, 36.0)	< 0.01*
Duration of surgery (mins) (Mean ± SD)	92.0 ± 45.0	91.8 ± 45.9	92.6 ± 43.4	*0.81*
Transfusion (ml)				
Blood (Mean ± SD)	7.1 ± 12.7	7.7 ± 13.8	5.7 ± 10.4	*0.52*
Plasma (Mean ± SD)	0.9 ± 1.8	1.4 ± 2.7	0 ± 0	*0.17*
Blood loss during surgery (ml) (Mean ± SD)	4.4 ± 4.0	4.3 ± 4.0	4.4 ± 4.1	*0.94*
Fluid infusion during surgery (mL) (Mean ± SD)	98.9 ± 50.8	99.9 ± 53.4	97.1 ± 44.8	*0.72*
Urine (ml) (Mean ± SD)	13.6 ± 11.0	13.2 ± 10.4	14.3 ± 12.2	*0.85*
Preoperative Hemoglobin (g/dL) (Mean ± SD)	146.3 ± 47.2	141.4 ± 41.3	156.9 ± 60.0	*0.39*
Postoperative Hemoglobin (g/dL) (Mean ± SD)	125.4 ± 34.2	124.8 ± 35.5	126.7 ± 31.2	*0.30*
Postoperative hospital length-of- stay (PLOS), days (Mean ± SD)	22.9 ± 18.6	23.9 ± 19.6	20.8 ± 16.6	*0.13*

Note: Sample (n), Standard deviation (SD), Interquartile Range (IQR), *P<0.05

### Factors associated with intraoperative hypothermia

Multivariable logistic regression was performed using the presence of intraoperative hypothermia as the dependent variable (Table 3). This complete case analysis included 249 neonates. Among the pre-chosen predictors, the length of surgical time (adjusted odds ratio, 1.01[95% CI, 1.00 to 1.02]; *p* = 0.025) and fresh gas flow (adjusted odds ratio, 3.04 [95% CI, 1.45 to 6.39]; *p* = 0.003) were independently associated with the occurrence of intraoperative hypothermia.

**Table 3:** Results of Multivariable Logistic Regression with Occurrence of Intraoperative Hypothermia

Variable	Adjusted Odds Ratios	95%CI	*P* value
Age (days)	0.99	0.94, 1.05	*0.95*
Male sex (vs. Female)	1.11	0.54, 2.29	*0.77*
Weight at the surgery time (kg)	0.52	0.09, 2.9	*0.46*
Birth body weight (kg)	1.42	0.26, 7.83	*0.69*
Gestation days (days)	0.99	0.96, 1.02	*0.56*
The length of surgical time (min)	1.01	1, 1.02	0.025*
Fresh gas flow (L/min)	3.04	1.45, 6.39	0.003*

Note: Results of multivariable logistic regression using pre-chosen predictors forced into the model 95%; Confidence Intervals (95% CI)

*P<0.05 is statistically significant

## Discussion

In our study, low fresh gas flow anesthesia (≤ 1 L/min) for the neonates undergoing digestive surgery was associated to reduce the incidence of hypothermia. This method could shorten the length of hypothermia, as well as help to alleviate further decreasing of body temperature. Moreover, by multivariable logistic regression performed, we found that low fresh gas flow anesthesia and the length of surgical time were significantly associated with intraoperative hypothermia.

In this study, intraoperative hypothermia was defined as a core temperature (nasopharyngeal temperature) < 36 °C, in accordance with the definition of the National guideline in UK [13]. The overall incidence of hypothermia was about 81.9% in our study, which was like the result (83.3%) from a study by Lai [4]. Lai et al. also pointed that the incidence of hypothermia was higher in neonates, compared with infants, toddlers and children [4]. From above evidence, we confirmed that compared to other population, neonates had a faster body heat loss during the intraoperative period due to their physiological characteristics. To our knowledge, hypothermia should be prevented rather than treated after its occurrence. With the increasing awareness of the harms of intraoperative hypothermia, many ways had been proposed to prevent it from happening. In our institution, the strategies for avoiding hypothermia included utilizing anesthesia station with heat and humidity exchanger, warm air circulation, warm infusion fluids, and warm mattress pads and blankets, but the incidence of hypothermia was still high before September 2019 (87.6%), which indicated the available strategies were not enough. To date, some anesthesia providers attributed it to patients’ age, weight and different surgical procedures. Actually, in 2017, Engorn et al. did a retrospective research in neonates and revealed that gestation days, weight, type of surgery, sex, and length of surgery were not the risk factors for hypothermia except the thermoregulation interventions, which pointed that active and passive warming strategies played the pivotal role in maintaining normothermia [12]. A study with multiple logistic regression in Thailand showed that the following risk factors for core hypothermia included high ASA physical status and open surgery. Significant protective factor against core hypothermia was heavier body weight [14]. In our study, the enrolled patients were neonates who underwent digestive surgeries, all of whom had the above risk factors rather than the protective factor, which demonstrated the importance of this study.

Our study proved that the length of hypothermia in low fresh gas flow (≤ 1 L/min) group was less than in routine group and the value of lowest body temperature was higher. As far as we know, inhaled air was warmed and humidified in upper air ways in spontaneous breathing. During mechanical ventilation with tracheal tube, this mechanism was disturbed, and the air was inhaled to lower airway directly without being warmed and humidified. Ventilation with dry and cold gases would lead to a considerable loss of heat from airway. In the Dräger Primus breathing system, to reduce the loss of heat, the exhaled gases moved through the hotplate once before mixing with cold and dry fresh air [15]. In 2011, Castro et al. reported that insertion of a heat and moisture exchanger increased the temperatures of inhaled gas in adults [15]. Moreover, by comparing 1 L/min and 3 L/min fresh gas flow in a Dräger Primus anesthesia workstation, Bicalho et al. revealed that 1 L/min fresh gas flow provided better inhaled gas temperature conservation in children [16], which implied that different gas flow was potentially influence body temperature. However, this theory had not been proven in neonates. In our institution, Dräger Primus anesthesia workstation was used for neonates, but the incidence of hypothermia was still high. Two factors might explain the difficulties to sustain normothermia in neonate even though some active and passive tactics had been applied. First, neonates had a large body face ratio, thin skin, low fat content, which was inconducive to temperature preservation. Of course, this problem was compounded by the effects of anesthetic agents that inhibited central thermoregulation by interfering with these hypothalamic reflex responses. Moreover, non-shivering thermogenesis by metabolism of brown fat was limited in premature or critical neonates who were deficient in fat store. Second, the maintenance of body temperature depended on the balance of heat production and loss. According to our results, we had enough evidence to doubt that compared to other resource, the heat loss from respiratory tract due to high fresh gas flow might occupy a large proportion and 1 L/min fresh gas flow might be the critical value. The body temperature was hard to be maintained when the fresh gas flow exceeded 1 L/min since heat loss was beyond production, but this was only our suspicion and further studies were needed to verify it.

Previous studies had revealed that hypothermia was correlated with increased perioperative blood loss and rates of surgical wound infection, which might result in prolonged hospitalization and increased incidence of postoperative adverse events [17, 18]. In neonates, the literature about the relationship between hypothermia and prognosis was limited. Our study demonstrated that compared to routine group, the value of lowest temperature was higher, and the duration was less in low flow group, which indicated that low fresh gas flow could alleviate the severity of hypothermia. However, we did not find that fresh gas flow had a relationship with PLOS. The result should be interpreted cautiously because of the shortage of medical resources and insufficient economic development in our region. The guardians might withdraw the treatment because of heavy economic burden, which could artificially shorten length of hospital stay. Besides, other potentially relevant clinical outcomes should be examined in our study—like wound infections, but we could not collect the data about wound infections from our electronic patient registration system. Some surgeons might be reluctant to admit the fact that the wound was infected. Thus, the wound infections were not recorded properly in the chart, especially in the patients with mild wound infections.

### Limitations

There were some limitations that should be noted. (1) This was a retrospective study which was at risk of patient selection bias and measurement bias. To reduce the effect of those bias, multivariable regression was conducted to determine the independent association between exposure and outcome. (2) Baseline and preoperative temperature were unknown. When the patients had pre-existed hypothermia, the outcome might be biased. (3) The confidence interval for the “treatment effect” of fresh gas flow was wide, and that a larger sample size would be needed to better delineate the true impact. (4) Because of the shortage of medical resources and insufficient economic development in developing countries like China, most neonates did not have medical-insurance, or the health insurance did not cover newborns. Some critically ill neonates with surgical indications might not be able to receive surgical treatment due to family poverty. Even the patients underwent the surgery, the guardians might withdraw the treatment because of heavy economic burden. In our study, the result of postoperative mortality and PLOS, therefore, might be biased. (5) One potential limitation of the investigation was limited generalizability to other settings.

## Conclusions

In summary, we concluded that low fresh gas flow (≤ 1 L/min) could be considered as an effective way to alleviate hypothermia for the neonates undergoing digestive surgery. Intraoperatively low fresh gas flow was independently associated with the occurrence of hypothermia.

### Ethic approval and consent to participate

Our study was approved by Institutional Review Board (IRB) at Chengdu Women’s and Children’s Central Hospital. After obtaining Institutional Review Board (IRB) approval [No. 2020(3)], we retrieved patient data from the electrical record system at Chengdu Women’s and Children’s Central Hospital.

## Supplementary information

**Additional file 1.**

## Data Availability

The datasets used and/or analysed during the current study available from the corresponding author on reasonable request.

## Abbreviations

PLOS: Postoperative hospital length-of- stay
IRB: Institutional Review Board
SD: Standard deviation
IQR: Interquartile range
