# Supplementary material for: The low fresh gas flow anesthesia and hypothermia in neonates undergoing digestive surgeries: a retrospective before-after study
Source: BMC Anesthesiol. 2020 Sep 3;20:223. doi: 10.1186/s12871-020-01140-5 (PMC7470439; doi:10.1186/s12871-020-01140-5)
Supplement: Supplementary file 1 — Additional file 1. [file 12871_2020_1140_MOESM1_ESM.docx]

**The main R packages were used in our study.**

| lattice | survival | Formula | carData | jmuOutlier | gvlma | plyr | rms | pastecs | abind |
| --- | --- | --- | --- | --- | --- | --- | --- | --- | --- |
| plotrix | car | ggplot2 | Hmisc | scales | Matrix | boot | nlme | tcltk | arm |
| RODBC | MASS | foreign | grid | SparseM | lme4 | MBESS | lsr | asbio | lubridate |
